# Travel medicine, coca and cocaine: demystifying and rehabilitating *Erythroxylum* – a comprehensive review

**DOI:** 10.1186/s40794-019-0095-7

**Published:** 2019-11-26

**Authors:** Irmgard Bauer

**Affiliations:** College of Healthcare Sciences, James Cook University, Townsville, QLD 4811 Australia

**Keywords:** Coca leaf, Acute mountain sickness, Narcotics, Cocaine, Altitude, Travel health advice

## Abstract

Few travel health measures are as controversial as the use of coca leaves at high altitude; yet, there appears widespread ignorance among health professionals and the general public about coca, its origins as well as its interesting and often flamboyant history. Equally, the cultural and traditional significance to Andean people is not recognised. The coca leaves contain many alkaloids, one of which, cocaine, has gained notoriety as a narcotic, leading to the mistaken idea that coca equals cocaine. This article contrasts coca with cocaine in an attempt to explain the differences but also the reasons for this widespread misconception. By its very nature, there may never be scientific ‘proof’ that coca leaves do or do not work for travellers at altitude, but at least a solid knowledge of coca, and how it differs from cocaine, provides a platform for informed opinions and appropriate critical views on the current confusing and contradictory legal situation.

*The use of the coca plant not only preserves the health of all who use it, but prolongs life to a very great old age and enables the coca eaters to perform prodigies of mental and physical labor.*

(John Pemberton, *Atlanta Constitution*, 1885)

## Introduction

Few travel health measures are as controversial as the use of coca leaves at high altitude. An informal brainstorming exercise among health professionals and members of the public yielded a number of reasons: cultural superiority, fear, unease, self-righteousness, but above all ignorance, confusion with cocoa, and the mistaken idea that coca equals cocaine. This paper aims to de-mystify the topic by informing about the leaf, its historical and cultural background, its physiological properties including its use at high altitude – where it crosses paths with travel medicine – as well as its progression to the enthusiastic welcome in the industrial world of the nineteenth century. By necessity, this article includes a summarised coverage of key aspects of cocaine to allow an objective assessment and the distinction between natural leaf and isolated alkaloid (and illegal drug) cocaine hydrochloride.

### Altitude tourism

The increasing popularity of adventure and sports tourism as well as better access to more remote areas see more travellers at ‘high altitude’ (commonly > 2500 m.a.s.l.). Peaks in the Himalayas (Pakistan, India, Nepal, China) rise to above 8000 m, in the Andes to above 6000 m, and over 500 peaks in the European Alps are higher than 3000 m. The North American Rocky Mountains are as popular as Mt Kenya (5199 m) or Mt Kilimanjaro (5895 m) in Africa or Aoraki/Mt Cook (3724 m) in New Zealand. With the melting of last remnants of snow at the previous ski resorts of Chacaltaya/Bolivia (5421 m) and Mérida/Venezuela (4767 m), the currently highest ski fields are in Breckenridge/Colorado (3914 m) and the Kleines Matterhorn in Zermatt/Switzerland (3883 m). Train lovers can ride the Qinghai-Tibet Railway (5072 m), the Ferrocarril Central Andino (4782 m) or the Argentinian Train to the Clouds (4220 m). Cities such as Lhasa (3650 m) or La Paz (3640 m/Airport El Alto 4061 m) add to the multiple opportunities of exposure to high altitude. Still missing from travel medicine discussions are skydivers who typically fly within minutes to jumping heights of around 4000 m and above. Anecdotal evidence suggests altitude symptoms that are separate from fear, panic or exhilaration.

### Altitude medicine

The first known mention of an altitude-related influence on humans and animals originates in the third decade BC in China [1]. In 1590, Jesuit José Acosta was the first to document graphically the symptoms of altitude sickness he suffered while crossing the Andes [2]. Potential positive effects of altitude on the human body have been explored, for example, in sports training camps [3]. However, the literature generally focuses on acute altitude mountain sickness (AMS), high altitude pulmonary oedema (HAPE) and high altitude cerebral oedema (HACE). Discussion on epidemiology, clinical presentation and pathophysiology is ubiquitous and can be obtained elsewhere. Important for this paper is the current knowledge base on prevention and treatment apart from general gradual ascent or rapid descent. The current gold standard is the use of acetazolamide even though its efficacy in preventing AMS incidents has been questioned [4, 5]. Elsewhere, it has been identified as carrying a higher risk for AMS [6]. Other substances are dexamethasone, ibuprofen, previously ridiculed Gingko biloba, caffeine [7] and many others. While pre-treatment with Gingko biloba (EGb761) prevented AMS [8] or reduced its severity [9], other results were indifferent [10, 11], with Gertsch [12] starting the experiment at a baseline at 4000 m which appears far too late in the ascent to draw meaningful conclusions. Too few studies employing different protocols, and different sources of preparation of this and other substances, point to the need for more rigorous investigations, yet, the methodological difficulties are formidable.

### Coca leaves and the Andean traveller

Virtually all travellers to the Andes will come across coca leaves and coca tea. The tea is served in eateries, trains and accommodations from the very basic to the most luxurious. People picking up arrivals at high altitude airports typically come armed with a hot thermos of coca tea to prevent *soroche* (AMS). Coca tea may come as a few hard, dry leaves in hot water looking pretty but doing little, or as commercially available teabags which, the leaves being chopped up, colour the hot water quickly; the slightly bitter taste is apparent, and an effect noticeable. Even better would be the chewing of the leaves though this is less popular with travellers. Some fanciful application in biscuits, ice cream or lollies have no effect. For many travellers, drinking coca tea is normal, others, due to ignorance, may feel like criminals [13]. For some it may be an exciting challenge [14], others understand coca as part of a cultural discourse with the local people [15]. Tourists visiting mines in Bolivia typically purchase coca with dynamite, fuses, alcohol and cigarettes as gifts for the miners to use and to offer to *El Tío* (the devil) for protection [16].

### Travel medicine research and coca

A number of reviews and guidelines provide recommendations for the prevention and treatment of AMS; some omit coca [17, 18], others point out that there are no efficacy studies of coca and coca products [19, 20], though calling coca ‘a weak cocaine’ [20] misses the mark.

A few descriptive studies include coca in their reports. A study of trekkers’ and guides’ knowledge of AMS prophylaxis in Nepal’s Everest region showed poor understanding of acetazolamide use [21], a finding also described elsewhere [22]. Trekkers also carried coca, among other preparations, but no further information is given. Post-trip questionnaires of 162 adults to Peru/Bolivia and Kenya/Tanzania regarding prevalence of and risk factors for AMS included a question on coca leaves in the South American group where almost 50% of all travellers used ‘coca leaves’, presumably because predominantly non-medical sources were consulted [6]. However, important information about the definition of ‘coca leaves’ is missing, e.g. chewing of dry leaves, dry leaves in hot infusions, infusions with teabags, as well as a plethora of factors that would make a link to AMS prevalence meaningful. Another attempt to gauge 100 travellers’ knowledge of AMS and its source was made in Cusco/Peru [23]. The questionnaire appears underdeveloped and poorly edited; the paper only describes that only 9% of respondents knew of acetazolamide’s preventative properties. As questions to prophylactic measures were open-ended, it is highly likely that many travellers will have mentioned coca. Not discussing the entire range of travellers’ chosen measures clearly is a missed opportunity for a balanced report. Another missed opportunity to gauge travellers’ coca use arises from a large (*n* = 5988) study on self-reported health problems among visitors to Cusco [24]. Altitude sickness prophylaxis had been prescribed pre-travel to 16% of travellers, one must assume acetazolamide. Altitude sickness was reported by 43% of travellers; 75% applied self-treatment (for all illnesses). Since that many treated themselves, it is highly likely that coca featured prominently. Detailed questions of type of preparation and use, in such a large sample, would have delivered helpful insight.

Another study in Cusco examined AMS impact on 991 travellers [25]. The use of coca products in pre-travel advice was recalled by over 5%; to prevent AMS, almost 63% used coca products, though different types of products with different efficacy, e.g. virtually none in lollies, were bundled together. The conclusion that the use of coca leaf product was associated with increased AMS frequency is not supported by the study design. It does not capture when and how coca was used, e.g. was it used before even the slighted symptoms appeared (such as on arrival), or was it used (long) after the onset of symptoms, when it cannot be called prevention anymore? How can one be certain what subjects meant in a questionnaire? Of course, people remember coca once they have symptoms; this is not prevention and leads to questionable conclusions.

One study attempted to describe 136 travellers’ use of coca in Peru and Bolivia with 15 closed questions [26]. Almost 90% took coca; of those, 55% drank coca tea, 22% chewed the leaves, and 23% did both. Forty-two percent wished to prevent AMS, 22% treated AMS, 36% cited other reasons. The reported effect is described but lacks meaning. Fifty-one percent reported no noticeable effect. Since there is no standardised dose, it is unclear how much was ingested. Apart from that, if taken for prevention and it worked – how would anyone know if it had or had not an effect – or if the individual simply had no symptoms? Thirty-one percent felt a desirable effect – again, how did they know that this was due to coca and not the excitement of the trip? Undesirable effects were reported by 19%, such as nausea and abdominal pain. There is no way travellers would have been able to specify that this was due to coca and not altitude, jetlag, food etc. The study listed a number of limitations correctly but was not able to add to the current limited knowledge. Finally, a ‘placebo-controlled, single-blinded, non-randomized’ study of homoeopathic coca use at Everest Base Camp described a significant reduction of the effects of altitude on a small sample of trekkers [27]. The study’s weak methodology is acknowledged but the paper omits one crucial part completely, the used homoeopathic preparation. On request, any disclosure of the nature of the compound or analysis was declined. For this reason, this paper should be excluded from any further discussions on the efficacy of coca.

### What is coca?

The coca shrub was named *Erythroxylum* by Browne in 1756 (*Erythroxylon* by Linneus in 1759) [28]. Over 200 different species of the bush can be found throughout South America, the Caribbean, Africa, India, tropical Asia and Oceania [29], not all containing alkaloids. The two species cultivated in South America are *E. coca* and *E. novogratense*, each with two variations adapted to different topography, soil conditions and climate. The best conditions are found in the subtropical valleys on the Andean Amazon slopes [30]. Apart from fibres, essential oils (flavour/aroma), minerals and vitamins, coca leaves contain generally a number (up to 15) of alkaloids in varying amounts, such as hygrine, cuscohygrine, *cis*- and *trans-*cinnamoylocaine, nicotine, methylecgomine, tropococaine, tropinone, benzoylecgonine, and cocaine [e.g. 31–34]. Because of the focus on cocaine, there has been little research into the properties of the leaf’s other alkaloids. This is disappointing as it is highly likely that the substances act synergistically to produce the effect of coca [35], and more research on the entire leaf is needed [36].

Whereas the individual alkaloids may be of interest to some only, the nutritional value of coca leaves with its relatively high levels of certain minerals and vitamins has been celebrated for decades after the famous ‘Harvard-Study’ [34]. Based on this analysis, the use of coca in food, particularly as coca flour, was promoted as a cheap local substance to improve the nutrition, especially of the poor. The study only advised caution regarding the alkaloids and possible insecticide residue. Later findings that coca had no nutritional benefits or even adverse effects were disappointing though the study acknowledged that there might be other factors affecting the bioavailability of nutrients [33]. One must be careful assigning coca as the cause of malnutrition when suppression of hunger is typically based on lack of sufficient food in the first place. Feeding people properly decreased chewing in an earlier experiment [37] but more work needs to be done to avoid pro-and anti-coca research bias.

Apart from the traditional topical application as powder or poultice, the hard sun-dried leaves are usually consumed as tea or by ‘chewing’. Chewing is not the right word as travelling novice chewers quickly find out. Chewing the leaves produces a non-enjoyable accumulation of stubborn small hard bits in one’s mouth. Rather, as Unanue [38] described, it is more of a ‘sucking’ (today, one is invited by locals to suck (*chupar*) coca). The leaves are placed one by one in the mouth, briefly broken up, and moved around with the tongue to form a quid that is then moved to the buccal cavity where it is gradually moistened by saliva. With a saliva-moistened stick, a little lime (*llipta*), e.g. ashes of burnt seashells, limestone, or plants but also soda bicarbonate, is added to enhance the effect though it is still unclear if this improves the flavour, promotes salivation, or releases alkaloids [39]. The ‘chewing’ then consists of sucking the copious saliva until the quid is spent. Travellers generally forgo the adjuvant. A chewer is recognised by the bulge on one side of the face rather like a qat chewer’s in Southern Arabia but that plant is entirely different (*Catha edulis*), and only fresh new growth is used. However, travellers to both locations will note a similar effect.

### Cultural significance, traditional use and a ‘sprinkling’ of research

Coca has always played an important role in Andean life [40] on two levels. First, coca is a strong marker of cultural identity for many Andean communities [41, 42]. It is crucial in acknowledging and maintaining social bonds [29]. Friendship and affection are demonstrated by chewing together; refusal is perceived antisocial [42]. Religious aspects of coca are evident through shamans’ divinations, at animal sacrifices and burials, but also in everyday life such as a gift to a potential bride’s father, during carnivals and celebrations, even as inspiration for weavers [42]. The chewing of a *cocada* is a measure of time indicating 45 min of walking comfortably for 2 km of steep terrain or 3 km of level grounds [42]. Strict etiquette rules the highly ceremonialised handling, sharing and use of the leaves [41]. Coca is part of important economic activities on a local level, e.g. rural women trading in traditional medicines [43], and nationwide [44].

Second, historically and today, coca is used for medicinal purposes. It works as a local anaesthetic in local applications (powder/poultice), by chewing against toothache or pain in mouth and throat, or as a tea for gastrointestinal complaints [30]; it eases the pain of childbirth and hastens labour [42]. Even today, Andean immigrants to the UK use coca, a cultural keystone species, in legal products, such as teabags, sweets and flour [45]. Historically, to facilitate work, coca was chewed three times per day, before starting, halfway through the day, and shortly before finishing [38]. Then and today, coca works as a stimulant, suppresses appetite and fatigue, and alleviates the effect of altitude [29]. These latter qualities created the enigma of coca and intermittent flurries of research, generally on high altitude residents, not new arrivals [46].

In the late 1940s and early ‘50s, research (using Western tests) linked coca chewing to mental deficiencies, low intelligence and unfavourable personality traits in indigenous people [37, 47]. Studies on coca appear to arrive in bursts, depending on prevailing worldviews. Renewed interest in the 1960s and ‘70s resulted in a number of haphazard studies on Andean residents to pinpoint, in typical reductionist fashion, what exactly caused the alleged ability to withstand cold, hunger and fatigue. A puzzling study on six coca chewing Machiguenga residents of the cloud forests (2300 m) led nowhere [48]. Coca chewing in the Peruvian village of Cachicoto (2400 m) was associated with inferior nutritional status, inferior personal hygiene, and increased hookworm anaemia, concluding that cocaine kept villagers in a permanent state of malnutrition [49]. At least, the researchers acknowledged the complexity and interaction of environmental and host factors. Residents of the Peruvian village Nuñoa (4000 m) assisted with the next three studies. One reported that coca (in contrast to imbibing alcohol) had no effect when exposing one foot to 0 °C for 1 hr (*n* = 29) [50]. No particular result was obtained from having six chewers and six non-chewers step on a box repeatedly for 10 min at different rates and box heights [51]. When 14 male villagers were exposed to 15.5 °C for 2 h wearing shorts and reclining on metal cots with a blanket, it was concluded that coca might conserve body heat [52]. The possible effects of coca chewing on erythropoiesis have been suggested [53]. An Amazon expedition in 1977 was the setting for the determination of the amount of cocaine in the blood of three Eurasians and one local [54].

Twenty years later, experimental evidence of 22 men from the Bolivian Altiplano (3800 m) suggested a physiological effect of coca chewing on their better ability to sustain strenuous work for 60 min [55] but not improved maximal exercise capacity or increased work efficiency [56]. Interestingly, results of a glucose tolerance experiment suggest that habitual coca chewers, due to an antagonistic action of coca metabolites on insulin, do not present hypoglycaemia caused by hypobaric hypoxia [57], a fact assumed 20 years earlier [58]. More recent work found no difference in aerobic capacity between chewers and non-chewers [59] but biochemical changes enhanced physiological performance at high altitude [60]. These authors point to the important distinction that this effect could be due to flavonoids in the leaf, not the extremely small amount of cocaine (chewing of 30 g of leaves resulted in 98 ng [= 0.000000098 g]) of cocaine [60]. That other alkaloids in the leaf are biologically active, e.g. reduce food consumption, has been demonstrated before [61]. None of those studies, of course, has considered possible hereditary factors that contribute to altitude adaptation in Andean residents [62], and how this influences any comparison with the effect of coca on travellers not benefitting from genetic variations through many generations raised at altitude. Finally, chewing did not induce genetic instability in cells on the oral cavity, and no adverse health effects in chewers were associated with DNA damage at moderate consumption [63].

### The history of coca

The history of the coca leaf is fascinating. For an exquisite and extraordinarily comprehensive discussion, the *History of Coca* by Mortimer MD [64], or its shorter version [65] is highly recommended. While his work ends with the beginning of the twentieth century, many sources mentioned below cover the remaining time very well.

#### From prehistory to the Spanish conquest

Historic summaries usually start with the first Spanish accounts of coca after the sixteenth century conquest. However, signs of coca cultivation date back to thousands of years, from Nicaragua to Chile [66]. Lime containers have been found from as early as 8000 BC [42]. Dental evidence and hair samples, together with mummies, ‘*coquero’* ceramics, burial offerings and signs of prehistoric lime production suggest that coca leaf chewing was relatively common from 2500 BC in Peru [67] and 1000 BC in Chile [29]. In some regions, a magical function of coca may have restricted its use to important persons in a social group [30]. Coca was used in highlands and lowlands alike [66].

The use of coca was long established when in the eleventh century the first Inca *Manco Capac* arrived to start a formidable empire. Several romantic myths tell of the sacred origin of the plant (*mama coca*) whose divine powers restricted its use to the royal élite (the descendants of the Sun god *Inti*) for religious, social and political purposes, though permission could be given to others as a special favour. Coca chewing by commoners could be punished severely but the use of leaves for offerings was permitted [30, 39]. Coca may have been used for medicinal purposes, such as for trepanations [39]. A Fray Tomás Ortíz is said to have mentioned coca first when travelling in Venezuela but Amerigo Vespucci’s letter of 1504 provides the possibly first description of the coca chewing technique and its effect against thirst [30].

A weakening Inca empire saw relaxed rules around the use of coca by the general public; with the conquest changing the entire political, social, cultural, religious and economic landscape after 1532, coca was chewed by all [30, 39]. Pizarro’s men mentioned coca and its special stores [68] but de la Vega [69], and later Unanue [38], reported the Spaniards’ prejudice and ignorance. The Spanish were in two minds about coca. The clergy and some conquistadors saw the ‘devil’s leaf’ [42] as a barrier to conversion and a symbol of persistent idolatry [37, 39], whereas the owners of mines and plantations gave coca to the miners, workers, runners, and porters to get maximum work output at minimum expense [30]. Duplicitous Philip II supported the supply of coca to workers but, at the same time, instructed missionaries to oppress its use [39]. Large coca production and trade began; many in Spain got very rich [30, 42]. The bishop of Cusco was a major coca dealer himself [46]. Indians from the highlands died not only from the well-known adverse effects of conquest and forced labour but, sent to work in plantations, succumbed to humid heat and tropical diseases [30, 70]. Few Spaniards chewed, some using sugar instead of *llipta* [38].

In 1653, Jesuit Bernabé Cobo was the first to mention the anaesthetic effect of coca against toothache following local advice not to pull his healthy tooth but chew coca instead [30]. In the eighteenth century, Antonio Julian suggested coca for the working classes in Spain to improve health and productivity, and as a cheap substitute for coffee and tea. Consequently, Spain could have a supply monopoly in Europe [39]. In his 1794 dissertation, Unanue cites a Doctor Don Pedro predicting times when there would be abundant trade in coca with the rest of the world [38]. He was right.

#### Coca in the industrial world

Having observed coca-chewers in South America, Italian neurologist Paolo Mantegazza returned to Italy in 1858 to recommend coca highly as an internal medicine based on his self-experiments. Coca was received enthusiastically and, by the end of the nineteenth century, many physicians prescribed the chewing of leaves as conducive to good health. By 1855, German chemist Friedrich Gaedcke had separated *erythoxylin* (=cocaine) from the leaf and in 1859, Albert Niemann isolated purified cocaine [42]. His student Wilhelm Lossen determined the molecular formula C_17_H_21_NO_4_ in 1865 [68]. From here on, the thousands of years old history of coca continues, and the now over 150-year-old history of cocaine begins, first as a natural component of the leaf, later as the isolated alkaloid. The excitement of the miraculous substance led to several simultaneous developments. No summary could do the enthusiastic descriptions of applications, (self-)observations and success stories by a multitude of medical doctors, scientists, and quacks justice, and the perusal of either originals or fascinating comprehensive historical reviews [eg. 39, 64, 65, 71–73] is highly recommended. In order to describe and explain coca, it is imperative to highlight the difference to cocaine. The literature on the following sub-sections’ themes is vast, educative and interesting. Space constraints only allow a severely summarised representation of key aspects.

#### The invigorating tonic

In 1863, the Corsican chemist Angelo Mariani produced the first stable preparation of coca by adding the extract to Bordeaux wine which not only made him fabulously wealthy but his many customers very happy. His ‘Vin Mariani’ sold all over the world as a ‘most efficacious tonic’. He also produced an elixir, pâte (Lozenges), pastilles and tea [74]. The wine received thousands (13 volumes in the British Library) of recommendations and endorsements, including from Popes Leo XIII, who drank copious amounts and awarded Mariani a Vatican medal, and Pius X, monarchs (Queen Victoria, Kings George I of Greece and Oscar II of Sweden/Norway), but also celebrities such as Thomas Alva Edison, Jules Verne, Eleonora Duse or Auguste Rodin [75]. It made people feel good, probably because Vin Mariani contained 0.12 grain cocaine per fluid ounce. One recommended glass of wine (1/2 glass for children) contained 6 fluid ounces [76] for a total ‘dose’ of 0.72 grain or 46.65 mg cocaine.

Mariani’s reputation was marred by inferior imitators, one of them a John Pemberton of Atlanta. Pemberton, who possibly hoped to break his morphine habit with coca, produced ‘French Wine Coca’, adding caffeine from the West African kola nut. He announced endorsements ‘by over 20,000 of the most learned and scientific medical men in the world’ [76, p.23]. Prohibition hampered his success, and the alcohol had to be removed from the beverage. Substitution with water was unsatisfactory but when mixed with soda water by lucky mistake, a drink was created that would conquer the world: Coca-Cola. Alleged crimes by black men against white women focused the investigation on the cocaine content of the drink which, reportedly, had been removed by 1903, an action that haunts the company to this day, stubbornly sticking to strategic untruths. Perused by business students around the world, the history of the beverage and of the company’s aggressive business practices, including child labour and rampant racism, make for riveting reading [76, 77], especially for health professionals. Remarkable is that, to this day, the presence of cocaine in Coca-Cola *ever* is being denied, and much is spent on ‘myth busting’ advertisements. In 2009, Coca-Cola was forced to issue corrective advertisements in Australian newspapers. The rectification of the denial of the historic cocaine content was conveniently ‘forgotten’ [78]. The company has been on the brink of disaster many times afterwards. Starting off as a refreshing ‘pick-me-up’, the beverage’s ingredients kept infuriating, from alcohol and cocaine to ‘teeth-rotting acid’ and obesity-inducing sugar. It is equally remarkable that there seem few independent publications on the company or transparency on the Stepan-Company which is said to import, with special permission, coca leaves and decocainise them for Coca-Cola. A statement that Coca-Cola ceased using coca for flavouring in 2000 [79] could not be verified.

#### Coca and the scientific community

Ubiquitous publications in the 1800s by medical doctors and others praised the use of coca (and its cocaine) for its most marvellous ability to alleviate or cure all manners of afflictions. Among those were asthma, colds, eczema, hysteria, melancholy, exhaustion, overwork, ‘affliction of timidity in society’, weak nervous systems, depression, digestive disorders, opium habit and alcoholism. On the plus side, coca increased and strengthened pulse, respiration, and urinary excretion, supported sexual activity, provided rosy cheeks and a deeply joyful sensation of feeling intensively alive, not to mention a stimulating effect on doctor and patient. The following, published in the *Lancet* and the *BMJ,* rely on self-experimentation, popular at the time, and minute recording of details. Bennett [80] was disappointed to notice no particular effect of coca on himself and rather proceeded to investigate cocaine, theine, caffeine, guaranine and theobromine in ‘lower animals’. Historical descriptions from the conquest to nineteenth century explorers’ dismissive accounts, such as by Poeppig and von Tschudi (whose attacks on coca Freud called slanderous), piqued the interest of two other scientists, though one [81, 82] could not get the desired result.

Seventy-eight year old Sir Christinson, on the other hand, extending his experiments to his students and his own sons, could not praise the benefits of coca enough [83]. He tested the alleged fatigue and hunger alleviating properties elaborately and extensively. Chewing specific amounts of leaves, he felt nothing until he left the house and started walking 16 miles. He ‘at once … was surprised to find that all sense of weariness had entirely fled and that [he] could proceed not only with ease but with elasticity’ (p.530). He had abstained from food and drink for 9 hrs, felt neither hunger nor thirst on arrival at home but ‘on dinner appearing in half an hour, ample justice was done to it’ (p.530). He concluded that chewing coca removed extreme fatigue and prevented it, hunger and thirst are suspended but appetite and digestion remain unaffected. He suggested a more palatable preparation such as a liqueur that pharmaceutical chemists could work out ‘it may be hoped, without looking for a patent’ (p.531). Coca’s wonderful properties also led to recommendations for tiresome travels [84] and, more recently, naturally occurring cocaine in the leaf has been found to possess insecticidal properties [85].

#### Cocaine as medicine

The application of patent medicines and tonics directed much work on the isolated alkaloid though still often called ‘coca’, a confusing mistake that persists even today. Sigmund Freud’s 1884 paper *‘Über Coca’* [86] praised the impressive qualities of cocaine. He prescribed it liberally to patients and to himself (to overcome his morphine addiction) and studied therapeutic outcomes including aphrodisiac effects, frightening his fiancée with the warning to expect ‘a big wild man with cocaine in his blood’. Accused of having added cocaine to morphine and alcohol as the third scourge of humanity, he stopped publishing on cocaine in 1887 [87].

At precisely the same time as Freud became famous, Alfredo Bignon, a French pharmacist in Lima, conducted numerous experiments on coca and cocaine but is today entirely ignored and forgotten. His contribution to the field is remarkable including his experiments with kerosene-precipitation to produce cocaine from coca, ‘the Bignon-Process’, a procedure that will surface again later in this paper. His method started the legal cocaine boom in Peru. He moved the production to the idyllic Tyrolian village of Pozuzo in the Amazon region and sent non-perishable cocaine sulphides to Germany, predominantly Merck, instead of bulky volumes of perishable leaves. This convenience resulted in the German dominance in science and global marketing of cocaine for decades [73].

Also in 1884, Freud’s friend, the ophthalmologist Karl Koller, experimented with cocaine as a local anaesthetic; its success extended later to dentistry, urology, laryngology and other fields, while William Halsted, also in 1884, developed a successful nerve blocking technique [68]. Like many other physicians who prescribed cocaine enthusiastically to their patients, families, friends and themselves, Freud and Halsted joined those who succumbed to the addictive effects of cocaine. The growing fear of cocaine, unfortunately, also changed the attitude towards coca [72]. A revival of the medical use of coca has been suggested in the 1970s as the abuse potential in coca leaves is low compared to the isolated alkaloid [88]. The clinical effects of coca and cocaine must be kept distinct, e.g. a cocaine user would consume 60% pure alkaloid nasally or intravenously immediately vs a coca chewer obtaining 0.5% cocaine in gradual release over 1 h [35]. More recently, coca chewing as a therapy for cocaine maintenance has been discussed [89].

#### Cocaine, the illegal drug

‘Cocaine is a tropane alkaloid that biologically acts as a serotonin-norepinephrine-dopamine reuptake inhibitor’ [36, p.568]. When the potential for addiction was understood, cocaine became illegal in the US in 1914 and its use declined. In the 1960s, cocaine use by the rich and famous encouraged millions to try this expensive and presumed ‘safer’ drug. The intense enjoyment of cocaine euphoria leads eventually to a loss of control over ‘intake and destructive behaviour of procurement … risking death, medical complications, incarceration, job loss, financial ruin and family turmoil’ [90, p.112]. Denial of this loss of control prevents addicts from seeking treatment. Cocaine dependence involves the brain’s regions involving reward and motivation, hence perpetuating the addiction [90].

##### Cocaine production

Today’s cocaine production is based on Bignon’s process mentioned earlier and starts in clandestine ‘laboratories’ with rudimentary equipment, such as gasoline drums and plastic lined maceration pits. Regardless of the method, the elaborate process involves the use of, among others, kerosene/gasoline/diesel, sulphuric acid, lime or caustic soda and much filtering, heating, neutralizing, and stomping, exposing farmers to great physical harm. At the end of the process, about 200-800 kg of leaves turn into 1 kg *pasta básica* [46, 91] which is then sent away for further chemical processes to become cocaine hydrochloride powder, ready for use. According to the Global Drug Survey 2018 [92], cocaine prices in New Zealand and Australian are the highest in the world at over A$ 300/g, compared to €60–80/g in Europe and less in South America. Thirteen percent of Australians surveyed could procure the drug within 30 min. Of great concern is the availability of ‘crack’, a cheap, highly addictive free-base form of cocaine, making the drug available to a much larger clientele. Cocaine production requires a vast amount of leaves and a solid knowledge of chemistry regardless of available YouTube instructions. Appreciating this process makes the often-mentioned rationale for banning the import of coca leaf teabags quite absurd. To make one’s own 1 kg of *pasta básica*, upwards of 800,000 teabags would be required. It is much easier to buy the product.

##### The legal situation

After cocaine was declared illegal in the US in 1914, a string of International Conventions on Narcotic Drugs followed in 1925 banning import/export and in 1931 limiting manufacturing and distributing narcotic drugs. In 1935, the US Narcotics Bureau ruled illegal the export of “Merchandise No. 5” (= coca and kola extract) which, with monetary persuasion, Coca-Cola managed to reverse in 1937 [76]. On instigation from Peru, after a brief fieldwork in Bolivia and Peru in 1949, the UN Economic and Social Council (ECOSOC) published a report on the ‘coca-leaf problem’ [93]. This report has since been heavily criticised for a number of serious flaws including prejudiced methodology and racist overtones. The fieldwork consisted mainly of ‘conferences’ with a range of people to elicit a wide spectrum of personal opinions with anti-coca opinions recorded in extraordinary statements on the degenerated lazy Indian race. Coca was said to affect intelligence and personality leading to moral decay; people being dirty, smelly, and negligent underlined their social inferiority. To the Commission’s credit, the report offered some moderating comments on some extreme views. Yet, in the end, its recommendations sided with the available anti-coca leaf chewing literature and opinions, and advised to limit legal crops for medicine and science only, and destroy the rest. Complete suppression of chewing should be achieved in 15 years or fewer (p.96). Perplexingly, today’s international bans are still based on this report.

The 1961 Single Convention on Narcotic Drugs (UN) [94] (amended 1972, 1990, 1999) is still the current legal document. Article 26 prescribes the uprooting of all wild coca bushes as well as the destruction of illegally (= not for medicine/science) cultivated bushes. Article 27 permits the use of leaves as a flavouring agent (for Coca-Cola). Article 49 (e): coca leaf chewing must be abolished within 25 yrs. Coca leaf was (and still is) listed as ‘Drug included in Schedule I’ [94, 95].

The 1988 UN Convention against Illicit Traffic in Narcotic Drugs and Psychotropic Substances [96] made possession, purchase and cultivation of coca leaves a criminal offence (turning millions of traditional coca users into criminals). The famous WHO/UNICRI 1995 ‘Global study on cocaine use’ concluded that the ‘use of coca leaf did not lead to noticeable damage to mental and physical health, that the positive health effects of coca leaf chewing might be transferable from traditional settings to other countries and cultures, and that coca production provided financial benefits to peasants’ [97, p.223]. The publication of the study was blocked by the US in the Committee B: 6th meeting under the threat of withdrawing funds. The WHO in 2006 acknowledged traditional use for health benefits but added ‘however, there may be insufficient research data to prove that using coca leaf brings only health benefits and no negative health consequence …’ [98]. Clearly, nobody wants to put a foot wrong. Since 2000, Peru is the only country exporting coca leaf to the world market (133 tons annually), the US the only importing country for flavouring agents and manufacture of cocaine [99].

The legal situation around the coca leaf use is confusing and the conventions contradicting, especially in light of the 2007 UN Declaration on the Rights of Indigenous Peoples. In 2013, Bolivia won the case to recognise traditional coca use and registered reservations to exclude coca leaf from Schedule I despite the objections of the US and 14 other nations. The complete removal of the coca leaf from Schedule I has been advocated for, and options and solutions offered, for decades to no avail [79, 100–102]. In a legal case in 2017, a Colombian national in Spain (possibly a victim of profiling) won the right to import coca leaf powder [103]. Travellers need to decide for themselves since no guidance is available.

##### The ‘War on Drugs’

Confusing legislation and a growing cocaine problem in the US led the Bush administration to the ideology-driven ‘War on Drugs’. The aim was to eradicate supply so that demand would cease. At great cost and with military assistance, coca bushes were to be uprooted manually in Peru and Bolivia – which forbids explicitly the use of herbicide for that purpose – and by glyphosate (trade name Roundup) aerial spraying in Colombia [104]. Not only did this ‘war’ fail for various reasons [105–110], the chemical eradication in Colombia caused serious health and environmental problems even across the border to Ecuador [108, 111–114]. Glyphosate also destroyed legal food crops and forests, made the soil useless for proposed ‘alternative crops’ and poisoned waterways [108, 111, 114]. Despite overwhelming evidence, the US State Department denied any health impacts and blamed farmers for the spray damage [111, 114]. A US official advised the *NY Times* in 2000 that glyphosate is ‘less toxic than table salt or aspirin’ and the victim’s accounts were ‘scientifically impossible’ [114]. A report prepared for the Inter-America Drug Abuse Control Commission in 2005 [115] confirmed that glyphosate‘s potential risks on human health are ‘essentially negligible’. Today’s evidence is different.

Coca eradication without replacement for local income is futile. Alternative crops or other means of economic development can be successful with the right approach and community involvement [116, 117]. Unfortunately, as part of the ‘war’, ‘alternative development’ [110] was designed in boardrooms, heavy-handedly executed and, most importantly, excluded local community leaders – and failed [117]. The European Union, for example in Bolivia, worked with local government and the coca-growers’ union resulting in sustainable development and a reduction of drug-related production [117]. Alternative development is a promising approach with does not need to exclude the coca leaf. Apart from the traditional use, more research should explore its use in a variety of products [118], possibly an insecticide [85] or potential novel food products as has been suggested for agroforestry trees [119]. The legal situation around the coca leaf is outdated and unsatisfactory; drug policy reform is urgently needed. It is small wonder that this confusion has triggered research into cocaine content in urine – also as a warning to travellers.

### Travellers as drug ‘delinquents’?

The alkaloid content of coca leaves has met with widespread interest, up to the minute botanical details of its distribution throughout the leaf [31]. The mindset firmly placed on illegality, the possibility of innocents’ positive drug testing reduced one paper on travel medicine to only warn against prospective legal trouble without any further discussion of its use [120]; another mentioned this aspect briefly in its discussion [25]. However, a few studies attempted to examine the actual cocaine content of coca leaf teabags and possible benzoylecgonine (BE) excretion, a primary urinary metabolite of cocaine (Table 1). This interest, as the publication dates indicate, is not only linked to the coca leaf consumption of travellers. From 1983, Delisse, a commercial mate de coca teabag, sold as Inca Health Tea and produced by ENACO (National Enterprise of Coca, Peru) became a popular beverage in the US. Though marketed as decocainised, suspicion lead to several teabag studies that, due to their diverse designs, cannot be compared and are, therefore, presented individually in Table 1.

**Table 1:** Studies on cocaine content in coca tea and benzoylecgonine concentration in urine

Authors	Year	Preparation of infusion	Cocaine content of infusion	Urinalysis	Benzoylecgonine (BE) concentration in urine	Comments
Siegel et al. [121]	1986	*N* = 36?	HIT^*^: ~ 4.8 mg Mate de Coca: ~ 5.7 mg (= 0.13–0.68% as normally found in coca)			No evidence of ‘decocainisation’, No ill effect, no abuse
ElSohly et al. [122]	1986	*N* = 1 1 teabag HIT in 1 cup	2.15 mg	6 samples within 29 h	Total amount excreted after 29 h: 818.8 μg + 31.1% of ingested dose.	Consider HIT consumption when interpreting data
Ferreira-Engelke et al. [123]	1991	40 teabags of HIT - 20 teabag (1 teabag/cup/10 min - 20 teabags extracted in ethanol/15 min (method by Turner [124]	X ± SD 0.04% ± 0.017 0.33% ± 0.035			Not decocainised; If stored for long (e.g. 3 years) cocaine in brewed tea 0.006–0.004%, in ethanol extraction 0.06–0.05%
Jackson et al. [125]	1991	*N* = 4 males 1 cup of HIT	1.87 mg	Samples over 36 h	Maximum BE concentration 1.4–2.8 mg/l Total BE excretion in 36 h 1.05–1.45 mg (= 59–90% of ingested cocaine)	Consider HIT when interpreting data
Floren et al. [126]	1993	N = 2 1 cup/240 ml of tea brought from Bolivia	Technical problems prevented cocaine testing. Estimated 0.8 mg of BE (3.4 μg/ml)		In one subject 2608 ng/ml after 4 h; Both subjects negative after 24 h	Authors concerned about having had coca tea for 10 days in Bolivia. ‘Experiment’ poorly described. Emphasise ‘illegality’. ‘Decocainisation cannot be done’ 20-30 mg per line of cocaine
Jenkins et al. [32]	1996	*N* = 1 Coca teabags from 1) Peru and 2) Bolivia randomly selected weighed and tea prepared 1 bag in 180 ml deionized water at 94 °C. Infusion times: 6, 9, 12, 15 min Subject drank 1 cup Peruvian tea; same subject drank 1 cup Bolivian tea on separate occasion	2 methods of methanolic extraction for both sources Peruvian tea: 4.14 mg (range 3.40–4.76 mg) Bolivian tea: 4.29 mg (range 4.09–4.49 mg)	Samples over 48 h	BE in urine for at least 20 h	Steeping time increased cocaine content in Peruvian tea (3.94–5.88 mg), approx. 80% of available cocaine transferred to tea. Consider tea when interpreting data.
Turner et al. [127]	2005	N = 1 1 teabag of Mate de Coca from Peru in 250 ml boiling water Steeped for 25 min.	2.5 mg	Samples prior to consumption and 2, 5, 8, 15, 20, 21, 24, 43, 68 h after	Positive for BE 2–24 h after consumption	[IB: Nobody would steep tea for 25 min!] Consider tea when interpreting data in sports drug testing
Mazor et al. [128]	2006	*N* = 5 1 teabag of Peruvian coca tea in 8 fluid ounces. Steeped for 15 min Each volunteer drank 1 more cup than the previous (A = 1 cup; B = 2 cups, etc)		Samples prior and at 2, 12, 24, 36 h after ingestion	Mean BE concentration in all samples: 1777 ng/ml (95%CI: 1060–2495)	Consider tea when interpreting data.

*HIT = Health Inca Tea

One should pay attention to the measurement units (e.g. μg, ng). Although the pure cocaine content in a line of the illegal drug is hard to judge depending on the quality (20-30 mg have been suggested [126], up to 2000 mg/day in studies [129] to up to 10 g/day [54]), the amounts ingested via leaves are not comparable with those taken up in a cocaine ‘line’. Unless one is in competitive sports or due to drug testing for forensic or medical purposes, BE in urine is no reason not to use coca leaves. To discriminate between the use of coca leaves and manufactured cocaine, hair analysis may be useful [42, 130].

To put things in perspective, similar studies have been undertaken with poppy seeds (*Papaver somniferum L*.), a popular ingredient in a wide variety of food products, to determine morphine and codeine content in the seeds as well as metabolites in urine. Results found 2–294 μg/g/seed of morphine and 0.4–57.1 μg/g/seed of codeine [131, 132]; > 300 μg/L opiate in urine [131]. Another study reported highly differing morphine concentrations depending on variety and type of harvesting (0.5–294 mg/kg/seed) [133]. The inadvertent intoxication of an infant in 2005 in Germany triggered more comprehensive work [134] pointing to the loss of morphine during food processing. This led to banning imports of seeds from Australia containing high concentrations of morphine, and the advice against the consumption of large volumes of raw poppy seeds. Again, inadvertent positive drug testing [135], also in oral fluid [136], must be considered. In contrast to coca leaves, and despite minute amounts of morphine and codeine in poppy seeds, people eat them without any concern. Nobody calls for controlled cultivation of grapes to combat the enormous health and social cost of alcoholism. Tobacco is the single most preventable cause of death in the world [137], yet, tobacco cultivation appears mainly controlled for fiscal, not health reasons. It seems hypocritical to abandon reason over minute amounts of BE in urine when people have no trouble ingesting highly toxic substances, heavy metals, pesticides, herbicides or hormones in their daily food.

### The coca leaf: misunderstood and maligned

The coca leaf has been plagued by ignorance and prejudice since the conquest. In the nineteenth century, Peru’s potential benefit from coca as a keystone marker of nationality was made impossible by the increasing cultural divide and racial hierarchy between urban elites and the coca-chewing Indian majority. When, driven by intellectuals, the *indigenismo* movement arrived in the early twentieth century, coca was demonised as a ‘degenerating vice … poisoning … Peru’s *raza indigena*’ [73]. This relentless view guided many Peruvian researchers who formed a strong lobby around anti-coca crusader Dr. Carlos Gutierrez Noriega opposite the more moderate Dr. Carlos Monge [73] and influenced the infamous ECOSOC report. Peruvian upper and middle classes have generally viewed indigenous chewing with contempt [58, 73], an attitude that can still be observed today. Examining literature from within the US context, bias can often be spotted in the choice of topic, hypothesis, protocol, interpretation, and the continuous use of the same laden words such as ‘cocaism’ (vs cocainism), or ‘addicts’ [37, 138, 139], labelling chewing a scourge and (non-evangelised) chewers ‘infra-social’ [138]. The term ‘cocaism’, of course, plays into the hands of the uninformed for whom it is all the same. Biased is also the thesis that coca chewing leads to miserable living conditions [eg. 37], a potential association sold as cause-effect relationship. Indefensible are suggestions that coca affects a person’s intelligence [37, 47] and the demand for the eradication of coca leaf use without understanding, consideration and appreciation of the complex traditional and cultural context [41].

Equating coca leaves with the illegal drug cocaine is absurd and has been condemned on many fronts throughout the decades [64, 88, 89, 140]. According to Toyne, to transfer critical attitudes about cocaine to coca is inappropriate and irresponsible: ‘comparing the physiological effect of chewing naturally occurring coca leaves to snorting manufactured cocaine powder is like “comparing fire hoses with flame throwers” (Karch)’ [46, p.21]. Interestingly, it is precisely this equation (coca = cocaine) why coca is not used in Ecuador. A combination of colonial religious and civil prohibitions, a historical lack of a mining industry, and the wiping out of up to 95% of the indigenous population in some areas meant that knowledge and use of coca are virtually absent [141]. Depicting coca as a plant with psychedelic properties used by primitive man in primitive cultures with pernicious consequences [139] certainly helped. Today, a 1990 law (Title VI, article 38) prohibits cultivation, use, gathering, storage or transport of coca plant or parts for any purpose. The Ecuadorian public accepts that coca means cocaine and so quietly ratifies the status – yet, health shops sell coca teabags [141]. Today, ‘cocaism’ appears to have been replaced there by alcoholism [142].

### The problem with research

It is, of course, easy to criticise the shortcomings of research on coca and its physiological effect on humans in earlier times considering the changing worldviews, personal motivations, differing perceptions, and often crude research methods with basic equipment. Relating to the use of coca to prevent AMS in travellers, the lack of efficacy studies has been noted [6, 19, 20, 143]. However, to then conclude that coca ‘does not work’ is premature since we do not know, precisely because there are no such studies. It needs more than some half-hearted attempt to include coca in travel medicine research and then blaming the leaf for not working. Rigorous medical study is usually understood to be quantitative laboratory-based research or clinical trials. Unfortunately, transfer of such results to real life can be inconsequential. This is the main methodological problem of studying the effect of coca on travellers. For obvious reasons, such research would need to be conducted pre, peri and post trip, including on location at altitude [144, 145] but the core barrier is the travelling populations itself.

Each traveller is unique in his or her makeup of a myriad of physical, mental and medical variables when arriving at altitude. Additional confounders unique to the individual traveller are, for example, length of travel, time of day, level of stress (e.g. left after work or had a few days of rest before the trip, heavy luggage, missed flights), jetlag, physical and mental state, headache, too little/too much to eat or drink, anxiety regarding the planned trip, and many more. There is no way travellers could be matched into experimental and control groups in any meaningful way. Travellers could not even be their own control as with each arrival even at the same location, most if not all variables will have changed. This is important not only for any biochemical tests but because what travellers feel (before, after, or without coca) is subjective and escapes clinical measurement. Questionnaires and scales can produce crude rankings but not comparable precise results, no matter how hard one tries. Trembling knees when the plane door opens at *El Alto* or a ‘fuzzy head’ cannot be measured; travellers’ descriptions can be captured in qualitative approaches but not generalised as is desired in medical research. Furthermore, a most vexing issue with prevention is that one will never know if it worked because one will never know if one would have suffered a particular ill without the preventative measure.

If the prevention has not worked, i.e. the person does have symptoms regardless, one still does not know if the measure had been applied appropriately, early enough, and at the necessary strength. It is virtually impossible to study ‘what would have been?’ or ‘would it have made a difference?’. To claim coca has not worked because one still has headaches does not consider a string of reasons why this may be so. None of this makes coca a good topic for ‘rigorous’ research. At this stage, no research may be better than biased, ideology-driven, policy-influenced projects that confirm what many like to see confirmed.

## Conclusion

The use of coca leaves by travellers to prevent altitude-related symptoms in the Andes is widespread, yet meets with disapproval by the travel medicine community. This paper aimed not at making a strong case for the use of coca for this purpose; rather, it presented information on the plant, its origins and history through the times and its traditional and cultural value to the peoples in parts of South America. It then proceeded to the illicit drug cocaine by explaining its history, its production as well as the current confusing legal situation, and the failed ‘War on Drugs’.

The fanatic campaign against the coca leaf based on religious, racist or self-righteous fervour evident in many decades of the twentieth century must be understood within the context of those times, even if it may be hard to accept today. What should not be accepted today is that critical questioning and decision-making are still influenced and dictated by attitudes and laws that are based on those earlier concepts. Almost two decades into the twenty-first century, a more enlightened approach is called for. Ignorance makes people fearful; fear rarely leads to measured, well-founded decisions, but to misdirected obsession, zeal, and prejudice. People hear ‘coca’ and automatically think ‘cocaine’. Although scientific evidence supporting the use of coca would help many to step back and re-calibrate, it is highly unlikely that there will ever be strong research evidence for or against coca use in travellers. Travellers will use it regardless. If this paper has assisted in providing more knowledge about coca and the difference between the entire coca leaf with its minute amounts of alkaloids (one of which is cocaine) and the isolated illegally manufactured purified drug cocaine hydrochloride – at no fault of the leaf – it has met its goal. As Paracelsus said: ‘*Sola dosis facit venenum’*.
